# Reduced expression of glycolate oxidase leads to enhanced disease resistance in rice

**DOI:** 10.7717/peerj.28

**Published:** 2013-02-12

**Authors:** Mawsheng Chern, Wei Bai, Xuewei Chen, Patrick E. Canlas, Pamela C. Ronald

**Affiliations:** 1 Department of Plant Pathology, University of California Davis, California, USA; 2 College of Life Sciences, Inner Mongolia Agricultural University, Huhhot, China; 3 Rice Research Institute, Sichuan Agricultural University, Chengdu, Sichuan, China

**Keywords:** Photorespiration, Glycolate oxidase, Disease resistance, NH1, NPR1, Glutaredoxin, Hydrogen peroxide, WRKY45

## Abstract

Glycolate oxidase (GLO) is a key enzyme in photorespiration, catalyzing the oxidation of glycolate to glyoxylate. Arabidopsis GLO is required for nonhost defense responses to *Pseudomonas syringae* and for tobacco *Pto/AvrPto*-mediated defense responses. We previously described identification of rice GLO1 that interacts with a glutaredoxin protein, which in turn interacts with TGA transcription factors. TGA transcription factors are well known to participate in NPR1/NH1-mediated defense signaling, which is crucial to systemic acquired resistance in plants. Here we demonstrate that reduction of rice *GLO1* expression leads to enhanced resistance to *Xanthomonas oryzae* pv *oryzae (Xoo)*. Constitutive silencing of *GLO1* leads to programmed cell death, resulting in a lesion-mimic phenotype and lethality or reduced plant growth and development, consistent with previous reports. Inducible silencing of *GLO1*, employing a dexamethasone-GVG (Gal4 DNA binding domain-VP16 activation domain-glucocorticoid receptor fusion) inducible system, alleviates these detrimental effects. Silencing of *GLO1* results in enhanced resistance to *Xoo*, increased expression of defense regulators *NH1, NH3*, and *WRKY45*, and activation of *PR1* expression.

## Introduction

Photorespiration, which is metabolically coupled with photosynthetic CO_2_ assimilation (the Calvin cycle), is an intensively studied topic in plant biology. Glycolate oxidase (GLO) is a key enzyme in photorespiration, catalyzing the oxidation of glycolate to glyoxylate with an equal molar amount of H_2_O_2_ produced (Foyer et al., 2009). Photorespiration counters the carbon fixation reaction of the Calvin cycle in term of its release of CO_2_ and can account for more than 20% loss of net CO_2_ assimilation in C3 plants (Peterson, 1983; Sharkey, 1988). However, photorespiration is believed to play various roles in plants despite this negative impact. In addition to the ancillary metabolic role in converting 2-phosphoglycolate to 3-phosphoglycerate as a carbon recovery system (Boldt et al., 2005), photorespiration has been suggested to play a role in amino acid metabolism (Keys, 2006), nitrate reduction (Rachmilevitch, Cousins & Bloom, 2004), stress resistance (Moreno, Martin & Castresana, 2005), and signal transduction (Verslues, Kim & Zhu, 2007).

The hydrogen peroxide produced by GLO during photorespiration can perturb the redox states of leaf antioxidant pools, leading to modified gene expression (Noctor et al., 2002). In particular, most attention has focused on the potentially damaging consequences of enhanced chloroplastic production of hydrogen peroxide in stress conditions, such as drought. It has been reported that high glycolate oxidase activity is required for survival of maize in normal air because a maize mutation in the *glycolate oxidase 1* gene conditioned a seedling lethal phenotype (Zelitch et al., 2009).

Hydrogen peroxide is thought to be a signaling molecule that activates plant defense response (Chamnongpol et al., 1998). For many years, NADPH oxidases and peroxidases were believed to be the major enzymes that produce hydrogen peroxide directly associated with hypersensitive responses (Bestwick et al., 1997), an acute programmed cell death response induced by pathogen infection. However, suppression of *GLO* genes may lead to lower production of hydrogen peroxide. Rojas et al. (2012) recently reported that GLO is required in the elicitation of the hypersensitive response observed during Pto-AvrPto-mediated and INF1 (a pathogen associated molecular pattern, or PAMP)-mediated defense responses in *Nicotiana benthamiana* and also in nonhost resistance to *Pseudomonas syringae*. The positive role of GLO in nonhost defense responses was also observed in Arabidopsis and was shown to be independent of NADPH oxidase activity (Rojas et al., 2012; Rojas & Mysore, 2012).

Four *GLO* genes are present in the rice genome and five GLO isozymes are expressed in rice leaves (Zhang et al., 2012). Zhang et al.reported that *GLO1* and *GLO4* are predominantly expressed in rice leaves, while *GLO3* and *GLO5* are mainly expressed in the root. When either *GLO1* or *GLO4* was silenced, expression of both genes were simultaneously suppressed and most of the GLO activities were lost in leaves, suggesting that GLO1 and GLO4 are the major contributors to the GLO activity in rice and their expression is tightly coordinated (Zhang et al., 2012). Two of the five isozymes are shown to be homo-oligomers composed of either GLO1 or GLO4 and the other three are hetero-oligomers composed of GLO1 and GLO4, implicating potential novel roles for GLO in rice (Zhang et al., 2012).

Rice GLO1 was identified among the 100 interacting proteins constituting a rice interactome involved in biotic and abiotic stresses (Seo et al., 2011). GLO1 interacts with a glutaredoxin protein, which interacts with TGA transcription factors. TGA transcription factors are well-known mediators of NPR1-regulated systemic acquired resistance (SAR). Rice NPR1-like protein NH1 interacts with TGA transcription factors and the “Negative Regulator of Disease Resistance” protein (NRR), which compromises resistance to *Xanthomonas oryzae* pv. *oryzae* (*Xoo*) when over-expressed in rice (Chern et al., 2005a; Chern et al., 2005b; Seo et al., 2011). NH1 and NH3 are key regulators of defense response in rice that, when expressed at elevated levels, enhance resistance to *Xoo*, the causal agent of the rice bacterial blight disease (Chern et al., 2005b; Bai et al., 2011). NH1 and NH3 mediate benzothiadiazole (BTH; a plant defense activator) induction of defense response in rice (Chern et al., 2005b; Bai et al., 2011). BTH also induces expression of the WRKY45 transcription factor, which plays a crucial role in BTH-inducible resistance to *Magnaporthe grisea*, the causal agent of the rice blast disease (Shimono et al., 2007).

These reports and observations led us to hypothesize that GLO1 may be involved in response to biotic stress in rice. To test if GLO1 is actually involved in disease resistance response, we silenced the *GLO1* gene in rice, using either a constitutive or an inducible promoter, and challenged the transgenic rice plants with Xoo. We found that transgenic rice silenced for *GLO1* are more resistant to *Xoo*. Real time RT–PCR results confirm silence of the *GLO1* gene and activation of defense associated genes.

## Materials and Methods

### Plant materials

The Liaogeng (LG) and Kitaake japonica rice (*Oryza sativa* L) cultivars were used for this study. LG and Kitaake rice are both susceptible to the Philippine *Xoo* strain PXO99. Rice plants were grown in green houses at UC Davis at 27–32 °C under natural sunlight. For *Xoo* inoculation, 5–6 weeks old plants were transferred to a growth chamber and inoculated with PXO99 by the scissor-dip method (Kauffman et al., 1973). *Xoo* population measurements were conducted as described before (Chern et al., 2005b). Growth chambers were set at 28 °C with a day/night time cycle of 14 h/10 h.

### Gene isolation and plasmid construction

The rice *GLO1* gene was obtained from a yeast two-hybrid screen (Seo et al., 2011). The first 800 nt of the cDNA clone (named GIF15), which contained a full-length *GLO1* cDNA, was cut out from the pAD-Gal4 prey vector using EcoRI at the 5’ end and PstI internal in the *GLO1* cDNA. This excised fragment (ca. 800 bp) was purified and cloned into the PstI site in the pBlueScript II SK- vector by a three-piece ligation, together with the Xa21 intron (ca. 840 bp) precut with EcoRI at both ends. The Xa21 intron was used as a spacer to stabilize the construct. The Xa21 intron was originally amplified with primers Xa21int-1 (5’AAGTCGACGAATTCCAGGTCAGCAAGTCCTTCC) and Xa21int-2 (5’AAGTCGACGAATTCATACTCTGTTTGAGCAGGA) and cloned into pBlueScript II SK-. The resulting clone was named dsGLO1/SK. The dsGLO1 insert was excised with BamHI and KpnI and subcloned into the Ubi-C1300 binary vector (Chern et al., 2001), resulting in plasmid Ubi-dsGLO1.

For the dexamethasone (DEX)-inducible, GVG (Gal4 DNA binding domain-VP16 activation domain-glucocorticoid receptor fusion)-driven construct (Aoyama & Chua, 1997), the same dsGLO1 insert was excised with XhoI and SpeI and cloned into the TA7002 binary vector precut with the same enzymes, yielding construct GVG-dsGLO1.

### Treatment with a glucocorticoid

For induction, GVG-dsGLO1 and control Kitaake rice plants are foliar-sprayed with 100 µM DEX one day before leaf tissue collection or two days before Xoo inoculation. DEX was first dissolved in 1 mL of DMSO (dimethyl sulfoxide) then dissolved in 150 mL 0.05% Tween 20.

### RNA extraction, RNA blot analysis and real time RT–PCR

Leaf samples were frozen and kept at −80 °C until use. Total RNA was extracted using Trizol reagent (Sigma) with 1 mL of Trizol per half leaf. RNA was precipitated with isopropanol, rinsed with 70% ethanol, and air-dried. RNA was resuspended in 90 µL of RNase-free water and digested with DNase I in a volume of 100 µL. RNA was further extracted with 300 µL of Trizol to remove DNase I and purified with NucleoSpin RNA II columns (E&K Scientific). cDNA was synthesized using 3–5 µg of total RNA. Synthesized cDNA was treated with RNase H and RNase A and purified with a DNA clean & concentrator-5 kit (Zymo Research) before use.

RNA blot analysis followed the procedure described before (Chern et al., 2001). The 3’ fragment of GLO1 cDNA beyond the region used for silencing (see above) was used as probe in the RNA blot analysis of GLO1. Quantitative RT–PCR was carried out as described before (Bai et al., 2011). Real time PCR reactions were performed on a Bio-Rad CFX96 Real-Time System coupled to a C1000 Thermal Cycler (Bio-Rad). The actin transcript level was used as the reference in real time RT–PCR experiments. Rice actin real time PCR used primers Actin-QF (5’CAGCCACACTGTCCCCATCTA) and Actin-QR (5’AGCAAGGTCGAGACGAAGGA). For quantification of gene expression of individual GLO genes, gene-specific primers were designed for each GLO gene. GLO1 used primers GLO1-RT4 (5’CTCCTGCCTTGTGAACCCTG) and GLO1-RT5 (5’GTGCAGAAACTCAAAGATCTCTC). GLO2 used primers GLO2-RT4 (CTCTGCTCAAGTCATCACCA) and GLO2-RT5 (CCATTCTTCTTGATTGCATGGCT). GLO3 used primers GLO3-RT4 (5’CCATCTTCGCGCTGAGCTAG) and GLO3-RT5 (5’TGCTTGGTCATCACCCTCATC). GLO4 used GLO4-RT4 (TGTCCTCCATTTCTGCACTAC) and GLO4-RT5 (GGCATTGTTTTGATGGTTTGTGTG). NH1 RT PCR used primers NH1-RT3 (5’CTGATCCGGTTTCCCTCGGA) and NH1-RT4 (5’GACCTGTCATTCTCCTCCTTG). NH3 RT–PCR used NH3-RT3 (5’TGCTACACCTCTGCTGGTTGA) and NH3-RT4 (5’GACCAGCAAACTCTTGAGTTGAG). PR1a RT–PCR used primers PR1a-C1 (5’CGTCTTCATCACCTGCAACT) and PR1a-C2 (5’TGTCCATACATGCATAAACACG). WRKY45 RT–PCR used primers WK45-Q1 (5’GGACCAGGGCGATGTCACGT) and WK45-Q2 (5’TGTCCATCCATGATTCTTCGGTGA).

## Results

### Constitutive *GLO1* silencing leads to programmed cell death and reduced plant growth

To investigate the possible involvement of glycolate oxidase in rice development and immunity, we devised to reduce the expression of the GLO1 (Os03g0786100) gene using the RNA interference (RNAi) approach. We used the first 800 bp of the *GLO1* cDNA as the target to generate a construct with the intron fragment of the *Xa21* gene used as a spacer between two head-to head fragments of GLO1 cDNA that leads to production of an RNA forming a hairpin structure. This construct was placed under control of the strong, constitutive maize *Ubi-1* promoter (resulting construct Ubi-dsGLO1). This construct was used to transform rice cultivar Liaogeng (LG). We obtained many green, hygromycin-resistant calli that carried the Ubi-dsGLO1 transgene. Approximately 20 transgenic plantlets were regenerated. However, upon transfer to greenhouse, almost all of the transgenic seedlings became sick and their growth stalled. Within a few weeks, most died and no seeds were obtained. These results indicate that silencing the *GLO1* gene in rice is lethal under green house conditions (strong light). Only two lines eventually grew to mature plants and set fertile seeds.

**Figure 1:**
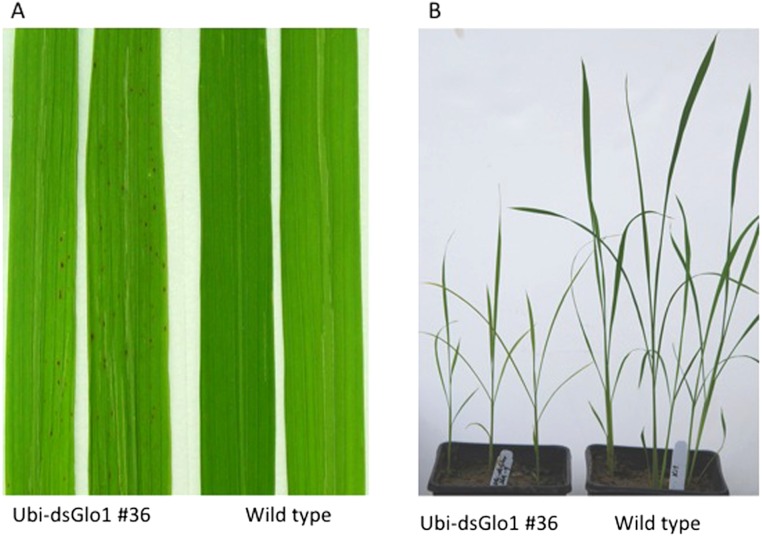
Lesion mimic phenotype and reduced plant growth of Ubi-dsGLO1 plants. (A) Lesion mimic phenotype. Two leaves from *GLO1*-silenced (Ubi-dsGLO1 line #36) and two leaves from wild type Liaogeng plants. (B) Reduced plant growth. Three weeks old plants of *GLO1*-silenced and wild type.

We observed lesion mimic phenotypes (results of programmed cell death) in the progeny of one of the two surviving lines that carry the Ubi-dsGLO1 construct. As shown in Fig. 1A, leaves of the progeny lines of Ubi-dsGLO1 line #36 developed tiny lesion mimic spots, characteristic of activation of defense response in plants (Chern et al., 2005b). The wild type LG plants grown together under the same conditions did not develop these lesion mimic spots.

We also observed a reduced plant growth phenotype in the Ubi-dsGLO1 progeny lines. As shown in Fig. 1B, three-week old seedlings of progeny of the Ubi-dsGLO1 #36 grow obviously smaller compared to the wild type plants of the same age when grown in a greenhouse together. Mature plants of Ubi-dsGLO1 plants also appear modestly smaller than wild type plants (not shown). This effect on plant growth has been observed repeatedly and consistently. Thus, constitutive silencing of *GLO1* appears to cause programmed cell death and reduced plant growth and development. These results are consistent with previous reports showing that *GLO* anti-sense rice grown under normal air developed growth retardation (Xu et al., 2009).

### Constitutive *GLO1* silencing results in enhanced resistance to *Xoo* and elevated *PR* gene expression

**Figure 2:**
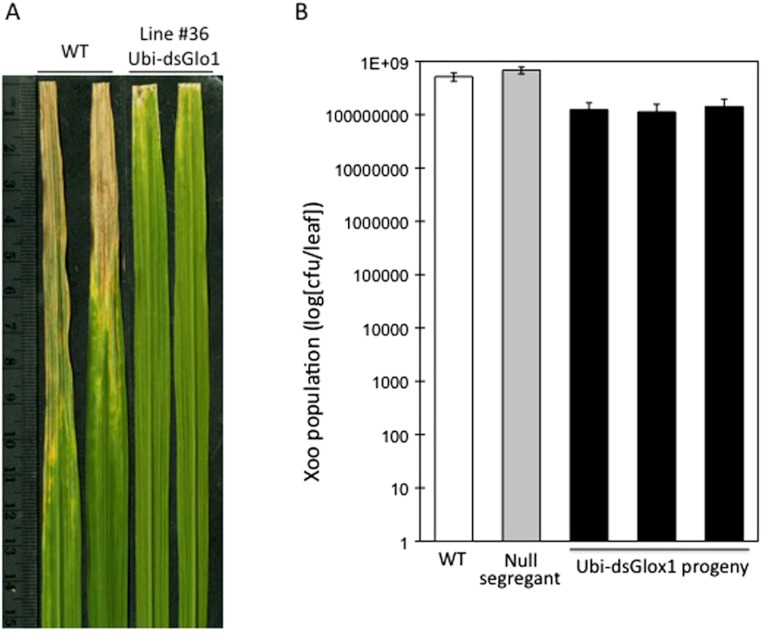
Enhanced disease resistance phenotype of Ubi-dsGLO1 plants. (A) *Xoo* induced disease lesions. Two leaves of *GLO1*-silenced (Ubi-dsGLO1 line #36) and wild type (WT) are displayed 2 weeks after *Xoo* inoculation. (B) *Xoo* populations. *Xoo* cells were extracted two weeks after inoculation and plated on medium containing 20 mg/L cephalexin. The bacterial population in each leaf was counted. Each bar represents the mean and standard deviation of three leaves.

To test the effects of *GLO* silencing on defense response, these two surviving lines were challenged with *Xoo* when they reached 6 weeks old. Rice cultivar LG is susceptible to *Xoo* Philippines race 6 (PXO99). Figure 2A shows two typical leaves two weeks after inoculation. Compared to wild type (WT), which developed lesions up to 10 cm, inoculated leaves of line #36 of Ubi-dsGLO1 developed lesions only 1–2 cm, indicating that this silencing line may harbor enhanced resistance to *Xoo*. The enhanced resistance phenotype was confirmed in the second generation when progeny of this line were inoculated with *Xoo*. *Xoo* populations of wild type, null segregants, and three lines of progeny carrying the transgene were determined. Figure 2B shows that progeny carrying the Ubi-dsGLO1 construct harbored significantly lower *Xoo* population (by about 8 × lower) compared to WT and null segregants. These results are consistent with the above observation that the Ubi-dsGLO1 plants possess enhanced resistance to Xoo, developing shorter lesions than wild type plants.

**Figure 3:**
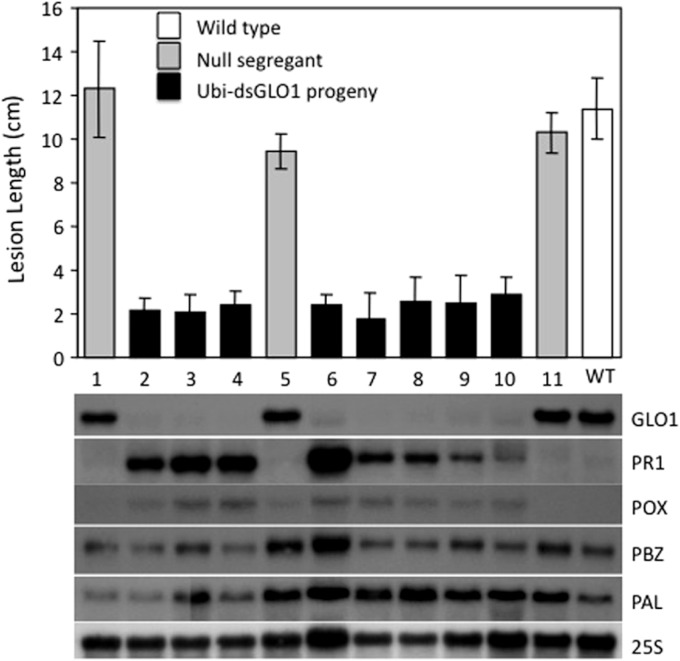
Segregation of resistance phenotype and RNA levels of *GLO1* and defense-related genes. Each plant (progeny of Ubi-dsGLO1 line #36) was genotyped for the presence of the Ubi-dsGLO1 transgene. Those containing the transgene are labeled Ubi-dsGLO1 (filled bars) and those missing the transgene labeled null segregants (grey bars). The *Xoo* induced lesion length of segregating progeny (labeled #1 to 11 under each bar) of Ubi-dsGLO1 and wild type LG (open bar) is plotted in the upper panel. The RNA levels detected by Northern analysis are displayed at the lower panel. The same progeny plant was used for analyses in the top panel and the bottom panel at the corresponding location. Each bar represents the mean and standard deviation of at least three leaves at the top panel.

We also analyzed the segregation of the transgene and the enhanced resistance phenotype among progeny of Ubi-dsGLO1 line #36. The upper panel of Fig. 3 shows that the enhanced resistance phenotype completely correlates with the presence of the transgene. All of the progeny that contain the transgene (filled bars) are resistant to *Xoo* and all null segregants (grey bars) behave like wild type (open bar) remaining susceptible to *Xoo*. We carried out RNA blot analysis to assess the expression levels of the *GLO1* gene and *PR* genes. The bottom panel of Fig. 3 shows that in the segregating population, the expression levels of *GLO1* are greatly reduced in the presence of the Ubi-dsGLO1 construct. The null segregants express the *GLO1* gene at levels similar to the WT. *PR1* is expressed at very low levels in WT and the null segregants, but expressed at elevated levels in the transgenic progeny lines. *Peroxidase* (*POX*22.3; Chittoor, Leach & White, 1997) RNA level, which is induced by Xoo infection, is also slightly elevated in the *GLO1*-silenced lines. *PBZ1* (probenazole-inducible) and *PAL* (phenylalanine ammonium lyase) expression levels were little affected in this experiment. These Northern results further show that Ubi-dsGLO1 line #36 carries reduced *GLO1* expression and elevated *PR1* and *POX* expression, confirming the activation of defense response in *GLO1*-silenced lines.

### Inducible silencing of *GLO1* achieves enhanced disease resistance with modest effects on plant growth

Constitutive silencing of *GLO1* results in activation of defense response and affects plant development. To alleviate these detrimental effects of constitutive silencing, we used an inducible promoter to drive the silencing construct. We employed a DEX-inducible, GVG-driven system (See Materials and Methods; Aoyama & Chua, 1997). The GLO1 silencing construct was placed behind a synthetic promoter whose expression is controlled by the GVG fusion protein, which requires activation by a glucocorticoid, such as DEX. This construct is named GVG-dsGLO1.

**Figure 4:**
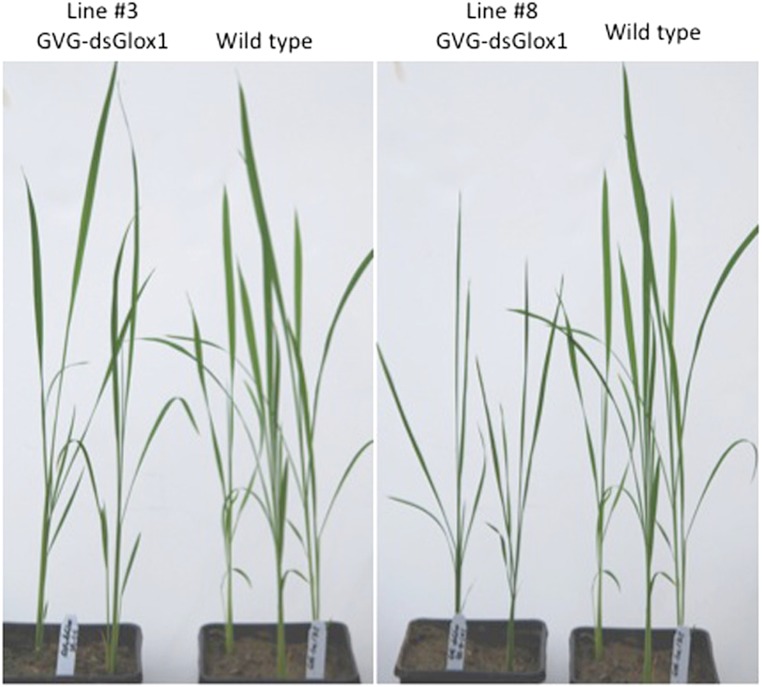
Morphology of three weeks old plants of GVG-dsGLO1 transgenics and wild type Kitaake control.

We generated approximately ten independent T0 lines harboring this GVG-dsGLO1 transgene. Figure 4 shows the growth of two independent (#3 and #8) GVG-dsGLO1 lines compared to a wild type control (Kitaake parent). Progeny of GVG-dsGLO1 line #3 show little difference from the control plants while progeny plants of line #8 show a moderate growth retardation phenotype. In addition, no lesion mimic phenotypes were observed in these transgenic lines. These results suggest that enhanced resistance may be achieved with little to no detrimental effects on growth by using an inducible promoter to control expression of the *GLO1* silencing construct.

**Figure 5:**
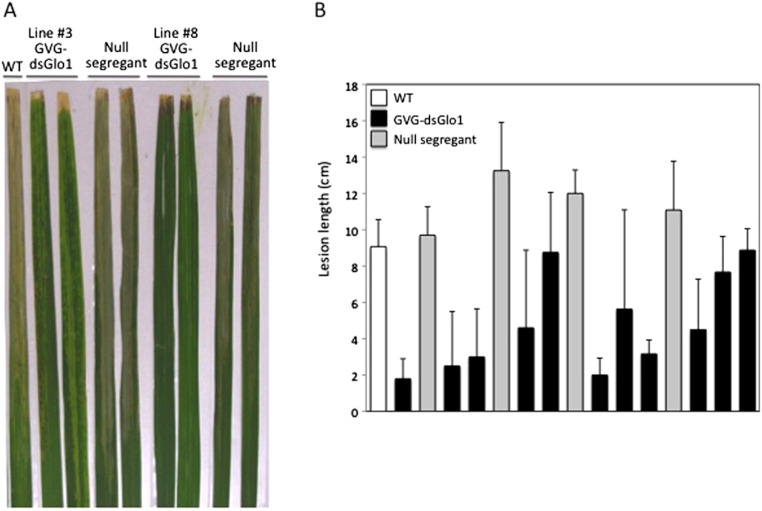
Resistance in GVG-dsGLO1 transgenic plants. All GVG-dsGLO1 plants plus null segregants and wild type control plants were sprayed with DEX two days before Xoo inoculation. (A) Two leaves each from GVG-dsGLO1 lines #3 and #8 and one leaf from wild type are displayed two weeks after *Xoo* inoculation. (B) Quantitative lesion lengths were measured for a segregating population of GVG-dsGLO1 line #3. Individual plant was genotyped for the presence of the transgene. Those containing the transgene are labeled GVG-dsGLO1 (filled bars) and those missing the transgene labeled null segregants (grey bars). Each bar represents the mean and standard deviation of three or more replicate leaves. A T-test analysis yielded a P value of < 0.0001 between the group of Ubi-dsGLO1 progeny and that of null segregants.

To test for enhanced resistance to Xoo, T1 progeny, instead of T0, plants were treated with DEX to avoid potential loss of transgenic lines due to detrimental effects after induction. T1 plants were treated with DEX and two days later inoculated with PXO99. Figure 5A shows leaves collected two weeks after *Xoo* inoculation and Fig. 5B shows segregation results of line #3. Wild type Kitaake (labeled WT) developed long (about 10 cm) disease lesion. Most of GVG-dsGLO1 line #3 transgenic plants developed shorter lesions whereas null segregants displayed long disease lesions. A T-test statistical analysis using all data of the line #3 transgenic progeny plants (group 1, *n* = 46, filled bars in Fig. 5B) compared with all data of the null segregants (group 2, *n* = 22, grey bars in Fig. 5B) gave a P value of less than 0.0001, demonstrating that the difference between the two groups is extremely statistically significant. The mean of group 1 is 4.8 cm (*S* *D* = 3.6; SEM = 0.5) while that of group 2 is 11.7 cm (*S* *D* = 2.1; SEM = 0.5). These results suggest that introduction of the GVG-dsGLO1 construct results in enhanced resistance to *Xoo*. The uneven lesion length distribution of the GVG-dsGLO1 progeny in Fig. 5B is likely due to uneven induction of silencing after DEX treatment. Overall, these results show that enhanced disease resistance can be achieved by silencing the *GLO1* gene in a controlled manner.

### Silence of *GLO1* leads to increased expression of defense regulators *NH1, NH3*, and *WRKY45*

**Figure 6:**
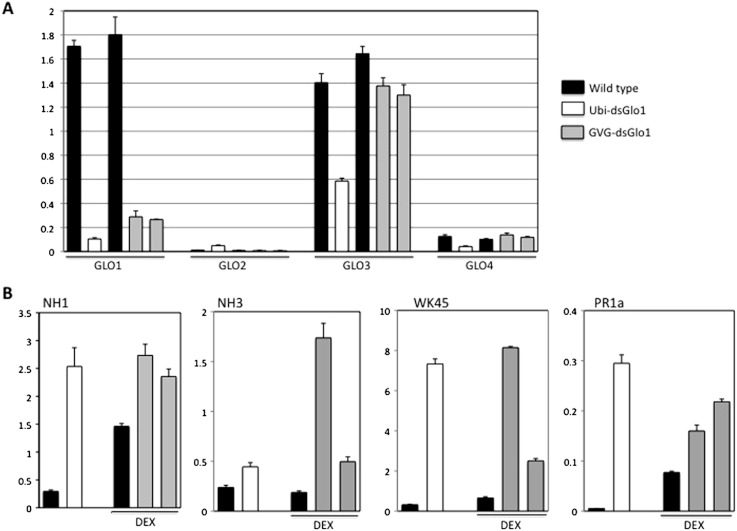
Real time RT–PCR of constitutive (Ubi-dsGLO1 line #36, open bars) and inducible (two progeny lines of GVG-dsGLO1 line #3, grey bars) silencing lines and their respective controls (LG and Kitaake). The GVG-dsGLO1 and Kitaake plants were sprayed with DEX one day before collection of leaf tissues for RNA extraction. (Ubi-dsGLO1 and its control were not treated with DEX.) In each panel, the first two bars represent data of Ubi-dsGLO1 and LG control and the next three bars represent data of GVG-dsGLO1 and Kitaake control. (A) Specific primers targeting each of *GLO1, GLO2, GLO3,* and *GLO4* were used for the four rice GLO genes to determine their RNA levels. (B) RNA levels of defense regulators *NH1*, *NH3,* and WRKY45, and defense marker gene PR1a were measured. Results were normalized to actin expression levels. Each bar represents the mean and standard deviation of three replicates.

To further characterize these *GLO1* silencing lines, real time RT–PCRs were performed to look at expression of *GLO* genes, *P*R1, and defense regulators *NH1*, *NH3*, and WRKY45 genes. Tissues of the GVG-dsGLO1 lines were harvested 24 h post DEX treatment. As shown in Fig. 6A, the level of *GLO1* expression in Ubi-dsGLO1 line #36 is (open bar) reduced by 16.4-fold compared to the wild type control (the first filled bar). The *GLO1* levels in GVG-dsGLO1 line #3 (two progeny lines are included, grey bars) are reduced by 6- to 7-fold compared to wild type plants (the second filled bar). Rice GLO1 cDNA shares the highest homology to GLO3 and the second highest to GLO4 at the nucleotide level. A nucleotide lineup is presented in Fig. S1. To assess possible effects on the RNA expression of other GLO genes, we also tested rice GLO2 (Os04g0623500), *GLO3* (Os07g0152900), and GLO4 (Os07g0616500) for their RNA expression levels. For direct comparison, results of all four GLO genes are combined in Fig. 6A. GLO2 is expressed at the lowest level of the four rice GLO genes in leaves. Surprisingly, rice *GLO3* is expressed at the second highest level – at a level similar to that of GLO1 in wild type rice. The RNA level of GLO3 is reduced by approximately 2-fold in the Ubi-dsGLO1 line (open bar) and only slightly reduced in the GVG-dsGLO1 lines (two grey bars compared to the filled bar immediately to the left). The RNA level of GLO4 is much lower than those of GLO1 and GLO3 genes (by 11- to 14-fold) in wild type rice measured in our real time PCR experiments. The RNA level of GLO4 in Ubi-dsGLO1 line is moderately reduced by approximately 3-fold compared to wild type, but those of GVG-dsGLO1 lines are unaffected. In summary, the RNA levels of GLO1 are greatly reduced in both Ubi-dsGLO1 and GVG-dsGLO1 lines, while those of GLO3 and GLO4 are modestly reduced in the Ubi-dsGLO1 line but not in the GVG-dsGLO1 lines.

Figure 6B presents the real time PCR results of defense related genes. The *PR1a* level in Ubi-dsGLO1 is elevated 55-fold in Ubi-dsGLO1 compared with wild type lines. The *PR1a* levels in GVG-dsGLO1 are only modestly elevated by 2- to 3-fold compared to wild type plants. The PR1a level in significantly elevated one day after the DEX treatment in kitaake plants compared to untreated Liaogeng plants; this may be due to the DEX treatment itself or may represent a difference between the two rice cultivars. Nevertheless, these real-time RT–PCR results are consistent with the Northern results shown above, confirming that, when *GLO* expression levels are reduced in Ubi-dsGLO1 plants, *PR*1a expression is greatly activated.

We also tested the expression levels of three key regulator genes (*NH1*, *NH3*, and WRKY45) of rice defense responses. Similar to Arabidopsis NPR1, NH1 interacts with TGA transcription factors, acts as a transcriptional co-activator, regulates *PR* gene expression, and contributes to innate immunity in rice (Chern et al., 2005b; Chern et al., 2012). NH3 contributes to BTH-inducible activation of rice defense response (Bai et al., 2011). WRKY45 is inducible by BTH-treatment and plays a crucial role in the BTH-inducible resistance to M. grisea (Shimono et al., 2007). The *NH1* level in Ubi-dsGLO1 (open bar) is greatly elevated by 8.5-fold compared to wild type (the first filled bar). The *NH1* levels in GVG-dsGLO1 (grey bars) are modestly elevated by 1.6- to 1.9-fold compared to wild type plants (the second filled bar) after DEX treatment. DEX treatment may also increase NH1 level in wild type. The *NH3* level in Ubi-dsGLO1 (open bar) is modestly increased by 2-fold compared to wild type. The *NH3* levels in GVG-dsGLO1 plants are increased by 2.6- to 9-fold compared to wild type. The WRKY45 RNA level in Ubi-dsGLO1 is highly elevated by 22-fold compared to wild type. The WRKY45 levels in GVG-dsGLO1 are also greatly elevated by approximately 4- to 12-fold compared to the DEX-treated wild type. The real time PCR experiment was repeated and similar results were obtained. These results show that reduction of rice GLO gene expression greatly induces expression of NH1 and WRKY45 and modestly induces expression of NH3. It should be noted that the smaller induction and greater variations in general in GVG-dsGLO1 plants 24 h after DEX treatment compared to Ubi-dsGLO1 plants may be simply due to sub-optimal induction at this time point. It is possible that greater induction may occur at other time points.

## Discussion

Our results show that reduction of *GLO1* expression in rice leads to enhanced resistance to the *Xoo* pathogen. When silencing the *GLO1* gene, the *GLO3* and GLO4 genes (the two most closely related rice paralogs) are moderately down regulated. *GLO1* and *GLO3* share 87% identity and GLO1 and GLO4 share 77% identify at the nucleotide level in the regions shown in Fig. S1. The probe (800 nt) used to silence *GLO1* probably contains identical regions large enough to affect *GLO3* and GLO4 RNA stability, resulting in moderate down regulation of the transcript levels of these two GLO genes in the Ubi-dsGLO1 line. However, the reduced GLO3 RNA level is much higher (5 × to 6 × ) than the reduced GLO1 RNA level in the Ubi-dsGLO1 line. Moreover, the RNA levels of GLO3 and GLO4 in the GVG-dsGLO1 lines are not (or little) affected after DEX-induction; by contrast, those of GLO1 in the GVG-dsGLO1 lines are greatly reduced. These results suggest that reduction of the GLO1 expression is the main cause of the activation of defense responses and enhanced resistance to Xoo, although contribution from reduced expression of GLO3 and GLO4 cannot be excluded at this point.

Zhang et al. (2012) reported that GLO1 and GLO4 are the two main GLO genes expressed in rice leaves. It is surprising to find that GLO3 is expressed at a level similar to that of GLO1 and much higher than that of GLO4 in our experiments. What caused the discrepancy is unclear. The primers that we used for the individual GLO genes in the real time RT–PCR experiments are unique to each gene. The fact that the reduced GLO3 expression level is much higher than that of reduced GLO1 in Ubi-dsGLO1 plants when normalized to actin expression suggests that the measured GLO3 levels are not results of cross-hybridization to GLO1 transcripts (which have the highest levels in wild type plants) by the GLO3 PCR primers.

It was shown that the hydrogen peroxide produced by GLO during photorespiration can lead to modified gene expression (Noctor et al., 2002). It has been reported that high glycolate oxidase activity is required for survival of maize in normal air (Zelitch et al., 2009). Photoinhibition was elicited when GLO reduction was over a threshold of 60% (Yamaguchi & Nishimura, 2000). We found that constitutive suppression of the rice *GLO1* gene over 60% (and partly of *GLO3* and *GLO4*) resulted in an evident lethal phenotype, probably a result of photoinhibition. Thus, our finding that constitutive silencing of GLO1 leads to a lethal phenotype is consistent with this report. Moreover, we have demonstrated that the use of a glucocorticoid-inducible promoter is able to alleviate this detrimental effect, but retain the enhanced resistance to *Xoo*. However, for practical applications, the timing of DEX application and the minimal effective DEX concentration needs to be determined. The duration of DEX induction is unknown.

Previous reports have shown that *GLO* activity is required in the elicitation of the hypersensitive response observed during nonhost defense and defense responses mediated by a disease resistance gene (Pto) or elicited by a PAMP (INF) in tobacco; *GLO* activity is also required in nonhost defense responses in Arabidopsis (Rojas et al., 2012). These reports suggest that reduced *GLO* expression would lead to lower hydrogen peroxide production, leading to lower defense response. In the current report, it is surprising to see that down regulation of *GLO* genes in rice resulted in enhanced resistance to the *Xoo* pathogen. It is likely that reduction of *GLO1* expression in rice may have induced basal resistance to a pathogen – a type of defense response different from those described above that are nonhost defense or effector- or PAMP-triggered defense responses (Rojas et al., 2012). It is also possible that GLO activity may play different roles regarding defense responses in monocots (such as rice) and in dicots (such as tobacco and Arabidopsis). In addition, our observation that GLO plays a negative role in defense is in line with our microarray data in which the components of photorespiration pathway were up-regulated in a super-susceptible transgenic rice line (NRR over-expression) (Chern et al., 2005a) compared to wild type (M Chern, KH Jung and PC Ronald, unpublished). One possibility is that the photorespiration pathway may be needed for *Xoo* pathogenesis.

Our RT–PCR analyses reveal another clue as to how reduction of GLO activity may lead to enhanced resistance to *Xoo*. The *NH1* and WRKY45 RNA levels are greatly elevated and the NH3 RNA level is moderately increased when *GLO* levels are reduced. Our previous reports have shown that elevated NH1 and NH3 levels both lead to enhanced resistance to *Xoo* and activation of *PR* gene expression (Chern et al., 2005a; Bai et al., 2011). WRKY45 is crucial to the BTH-inducible resistance to M. grisea (Shimono et al., 2007). NH1, NH3, and WRKY45 are key regulators of the BTH-mediated defense response in rice. Thus, it is likely that reduction in GLO activity may have led to activation of the BTH-inducible defense pathway, including elevation of the RNA expression levels of NH1, NH3, and WRKY45. The observed enhanced resistance to *Xoo* and activation of *PR* gene expression may be a direct consequence of the elevated levels of *NH1, NH3, and WRKY45* in *GLO1*-silenced plants. How reduced *GLO* levels lead to activation of this defense pathway remains a question to be answered.

## Supplemental Information

10.7717/peerj.28/supp-1 Fig. S1 The nucleotide lineup results of BLAST searches are displayed showing (A) the homology between *GLO1* and *GLO3* cDNAs and (B) the homology between *GLO1* and *GLO4* cDNAs.
