# Supplementary material for: Reduced expression of glycolate oxidase leads to enhanced disease resistance in rice
Source: PeerJ. 2013 Feb 12;1:e28. doi: 10.7717/peerj.28 (PMC3628735; doi:10.7717/peerj.28)
Supplement: Fig. S1 [file peerj-01-28-s001.pdf]

Figure S1

A. GLO1 vs GLO3

```
GLO1  132    GAAAATGGGGGAGATCACCAATGTCATGGAGTACCAGGCCATCGCGAAGCAGAAGCTTCC  191
             ||| |||||||||||||||||||||| |||||||||||||||||||||||||||||||||
GLO3  361    GAAGATGGGGGAGATCACCAATGTCACGGAGTACCAGGCCATCGCGAAGCAGAAGCTTCC  420

GLO1  192    GAAGATGATCTACGACTACTACGCCTCTGGAGCTGAGGATGAGTGGACTCTCAAGGAGAA  251
             ||||||||||||||||||||| || ||||| || ||||| |||||||||||| |||||||
GLO3  421    GAAGATGATCTACGACTACTATGCATCTGGGGCCGAGGACGAGTGGACTCTCCAGGAGAA  480

GLO1  252    CAGGGAGGCATTCTCCAGGATCTTGTTTCGTCCC-CGCATCCTGATCGATGTCTCAAAGA  310
             ||||||||| ||| |||||||| ||||||| ||| ||||| |||||||| || || ||||
GLO3  481    CAGGGAGGCCTTCGCCAGGATCCTGTTTCG-CCCACGCATACTGATCGACGTATCCAAGA  539

GLO1  311    TCGACATGTCCGCAACTGTTCTTGGGTTCAAGATATCAATGCCCATAATGATTGCTCCCA  370
             | |||||| || |||| || || ||||||||||| ||||||||||| |||||||||||| 
GLO3  540    TTGACATGGCCACAACAGTCCTGGGGTTCAAGATTTCAATGCCCATTATGATTGCTCCCT  599

GLO1  371    GTGCCATGCAGAAGATGGCTCACCCTGACGGAGAGTATGCAACAGC-CAGGGCAGCTTCA  429
              |||||||||||||||||||||||| || |||||||||||||| || | | || || |||
GLO3  600    CTGCCATGCAGAAGATGGCTCACCCAGATGGAGAGTATGCAACTGCGC-GTGCTGCATCA  658

GLO1  430    GCAGCAGGAACTATAATGACACTGTCATCTTGGGCTACTTCAAGTGTTGAGGAGGTCGCC  489
             ||||| || |||||||||||| ||||||| |||||||||||||| ||||||||||| |||
GLO3  659    GCAGCGGGCACTATAATGACATTGTCATCATGGGCTACTTCAAGCGTTGAGGAGGTTGCC  718

GLO1  490    TCAACTGGACCAGGAATCCGTTTCTTCCAGCTATACGTGTACAAGGACAGGAATGTGGTT  549
             |||||||||||||| || |||||||||||||| || || ||||| |||||||  ||||| 
GLO3  719    TCAACTGGACCAGGGATTCGTTTCTTCCAGCTTTATGTTTACAAAGACAGGAGGGTGGTG  778

GLO1  550    GAGCAGCTTGTCAGAAGAGCCGAGAGAGCTGGGTTCAAGGCGATCGCACTGACCGTGGAC  609
             ||||||||||| || ||||| || || ||||| ||||||||||| || || || ||||| 
GLO3  779    GAGCAGCTTGTTAGGAGAGCTGAAAGGGCTGGATTCAAGGCGATTGCGCTTACGGTGGAT  838

GLO1  610    ACTCCGCGACTGGGACGCAGGGAAGCTGACATCAAGAACAGGTTCGTTCTGCCACCATAC  669
             ||||||||||| || || ||||| |||||||||||||||||||| |||||||||||||  
GLO3  839    ACTCCGCGACTTGGCCGTAGGGAGGCTGACATCAAGAACAGGTTTGTTCTGCCACCATTT  898

GLO1  670    TTGACACTGAAGAA-TTTCGAAGGCCTGGACCTCGCAG-AGATGGACAAGAGC-AACGAT  726
             |||||||||||||| ||| ||||||||||| || |  | | |||||| || || | ||||
GLO3  899    TTGACACTGAAGAACTTT-GAAGGCCTGGAGCT-GGGGAAAATGGACCAG-GCTAGCGAT  955

GLO1  727    TCCGGGCTGGCCTCCTACGTCGCCGGGCAGATCGATCGCACGCTCAGCTGGAAGGATGTG  786
             || || ||||| || |||||||||||||| ||||||||||| || |||||||||||||||
GLO3  956    TCAGGACTGGCTTCTTACGTCGCCGGGCAAATCGATCGCACCCTGAGCTGGAAGGATGTG  1015

GLO1  787    AAGTGGCTGCAGAGCATCACGTCGCTGCCGATCCTCGTCAAGGGCGTCATCACCGCGGAG  846
             ||||||||||||| ||||||  ||||||||||||| || ||||| || ||||| || || 
GLO3  1016   AAGTGGCTGCAGACCATCACCACGCTGCCGATCCTGGTGAAGGGAGTGATCACTGCAGAA  1075

GLO1  847    GATGCGAGGCTGGCCGTGCACTCCGGCGCGGCGGGGATCATCGTGTCGAACCACGGCGCG  906
             ||  ||||||| |||||| |   ||||||||| || ||||||||||| ||||||||||| 
GLO3  1076   GACACGAGGCTCGCCGTGGAGAACGGCGCGGCCGGCATCATCGTGTCCAACCACGGCGCC  1135

GLO1  907    CGGCAGCTGGACTACGTCCCGGCGACCATCAGCGCCCTGGAGGAGGTGGTCACGGCGGCG  966
```

```
            || |||||||||||||||||||| |||||||||||||| |||||||| || | ||||||
GLO3  1136  CGCCAGCTGGACTACGTCCCGGCCACCATCAGCGCCCTCGAGGAGGTTGTGAAGGCGGC-  1194

GLO1  967   GCG-GGGC-GCATCCCGGTGTACCTCGACGGCGGCGTCCGCCGCGGCACCGACGTCTTCA  1024
             || |||| || |||| || | ||||||||||||||||||||||||||||||||||||||
GLO3  1195  TCGCGGGCAGC-TCCCCGTCTTCCTCGACGGCGGCGTCCGCCGCGGCACCGACGTCTTCA  1253

GLO1  1025  AGGCGCTCGCCCTCGGCGCCGCCGGCGTCTTCATCGGGAGGCCGGTGGTGTTCGCGCTGG  1084
            ||||||||||||||||||||||||||||||||||||||||||||||||||||| ||||||
GLO3  1254  AGGCGCTCGCCCTCGGCGCCGCCGGCGTCTTCATCGGGAGGCCGGTGGTGTTCTCGCTGG  1313

GLO1  1085  CGGCGGAGGGGGAGGCCGGGGTGAGGAACGTGCTGCGGATGATGCGGGAGGAGTTCGAGC  1144
            |||||| |||||||||||| ||  |||||||||||| |||| | || || ||||||||||
GLO3  1314  CGGCGGCGGGGGAGGCCGGAGTCCGGAACGTGCTGCAGATGCTCCGCGACGAGTTCGAGC  1373

GLO1  1145  TCACCATGGCGCTCAGCGGATGCACCTCGCTCGCCGACATCACCCGCGCCCACATC-TAC  1203
            ||||||||||||||||||| |||||||| ||||||||||||||||||  |||| || | |
GLO3  1374  TCACCATGGCGCTCAGCGGCTGCACCTCCCTCGCCGACATCACCCGCAACCACGTCAT-C  1432

GLO1  1204  ACCGACGCCGAC  1215
            ||||| ||||||
GLO3  1433  ACCGAGGCCGAC  1444
```

B. GLO1 vs GLO4

```
GLO1  775   TGGAAGGATGTGA-AGTGGCTGC-AGAGCATCACGTC-GC-TGCCGATC-CTCGTCAAGG  829
            ||||||||| | | ||||||| | ||  ||| ||  | || ||||||||  || ||||||
GLO4  711   TGGAAGGATAT-AGAGTGGCT-CAAGTCCATAAC--CAGCATGCCGATCTTTC-TCAAGG  765

GLO1  830   GCGTCATCACCGCGGAGGATGCGAGGCTG-GCCGTGCACTCCGGCGCGG-CGGGGATCAT  887
            || || ||||||| ||||| ||||||  | || ||| |  |||| | || ||| | | ||
GLO4  766   GCATCGTCACCGCTGAGGACGCGAGG-AGAGCAGTGGAGGCCGGGGTGGCCGGCG-TGAT  823

GLO1  888   CGTGTCGAACCACGGCGCGCGGCAGCTGGACTACGTC-CCGGCGACCATCAGC-GCCCTG  945
            ||| || |||||||||||||||||||||||||||| | |||||||||||| || ||||||
GLO4  824   CGTCTCCAACCACGGCGCGCGGCAGCTGGACTACG-CGCCGGCGACCATC-GCCGCCCTG  881

GLO1  946   GAGGAGGTGGTCACGGCGGCGGCGGGGCGCA-TCCCGGTG-TACCTCGACGGCGGCGTCC  1003
            || |||||||||| ||||| ||| || |||  | |||||| |   ||||||||||  |||
GLO4  882   GAAGAGGTGGTCAGGGCGGTGGCCGG-CGCCGTGCCGGTGCTGG-TCGACGGCGGAATCC  939

GLO1  1004  GCCGCGGCACCGACGTCTTCAAGGCGCTCGCCCTCGGCGC-CGCCGGCG-TCTTCATCGG  1061
            | || ||||| ||||| |||||||| || || |||||||| ||  |||| || |  | ||
GLO4  940   GGCGAGGCACTGACGTGTTCAAGGCCCTGGCGCTCGGCGCACG--GGCGGTCATGGTTGG  997

GLO1  1062  GAGGCCGGTGGTGTTCGCGCTGGCGGCGGAGGGGG-AGGCCGGGGTGAGGAACGTGCTGC  1120
            |||||||||| | |||| |||||||||| |||||| |||| || | |||| ||||| | |
GLO4  998   GAGGCCGGTGTTCTTCGGGCTGGCGGCG-AGGGGGGAGGCAGGCGCGAGGCACGTGAT-C  1055

GLO1  1121  G-GATGATG--CGGGAGGAGTTCGAGCTCACCATGGCGCTCAGCGGATGCACCTCGC-TC  1176
            | |||| |   |||  | || | ||| |  ||||||||||| |||| ||| |   || ||
GLO4  1056  GAGATGCTCAACGGC-G-AGCTGGAGGTGGCCATGGCGCTCTGCGGCTGC-CGGAGCGTC  1112

GLO1  1177  GCCGACATCACCCGC-GCC  1194
            | ||| ||||||||| |||
GLO4  1113  GGCGAGATCACCCGCAGCC  1131
```
